# The effect of a spontaneous induction prophage, phi458, on biofilm formation and virulence in avian pathogenic *Escherichia coli*

**DOI:** 10.3389/fmicb.2022.1049341

**Published:** 2022-11-14

**Authors:** Dezhi Li, Wei Liang, Qingyue Hu, Jianluan Ren, Feng Xue, Qing Liu, Fang Tang

**Affiliations:** ^1^School of Health Science and Engineering, University of Shanghai for Science and Technology, Shanghai, China; ^2^MOE Joint International Research Laboratory of Animal Health and Food Safety, Key Laboratory of Animal Bacteriology, Ministry of Agriculture, College of Veterinary Medicine, Nanjing Agricultural University, Nanjing, China; ^3^The Fourth Affiliated Hospital of Guangxi Medical University, Liuzhou, Guangxi, China

**Keywords:** avian pathogenic *Escherichia coli*, prophage, spontaneous induction, virulence, biofilm formation

## Abstract

Prophage sequences are present in most bacterial genomes and account for up to 20% of its host genome. Integration of temperate phages may have an impact on the expression of host genes, while some prophages could turn into the lytic cycle and affect bacterial host biological characteristics. We investigated the role of spontaneous induction prophages in avian pathogenic *Escherichia coli* (APEC), which is the causative agent of avian colibacillosis in poultry, and considered a potential zoonotic bacterium related to the fact it serves as an armory of extraintestinal pathogenic *E. coli*. We found that APEC strain DE458 had a high spontaneous induction rate *in vivo* and *in vitro*. The released phage particles, phi458, were isolated, purified, and sequenced, and the deletion mutant, DE458Δphi458, was constructed and characterized. Biofilm formation of DE458Δphi458 was strongly decreased compared to that of the wild-type strain (*p* < 0.01). In addition, while the addition of DNase (100 μg/ml) did not affect prophage release but could digest eDNA, it significantly reduced the biofilm production of DE458 biofilm to a level close to that of DE458Δphi458. Compared to DE458, the adhesion and invasion abilities of DE458Δphi458 increased by approximately 6–20 times (*p* < 0.05). The virulence of DE458Δphi458 was enhanced by approximately 10-fold in chickens based on a 50% lethal dose. Furthermore, avian infection assays showed that the bacterial loads of DE458Δphi458 in the lung and liver were increased by 16.5- and 10-fold (*p* < 0.05), respectively, compared with those of the WT strain. The qRT-PCR revealed that deletion of phi458 led to upregulation of type I fimbriate*-*related gene *fimH* and curli-related gene *csgC* by 3- and 2.8-fold, respectively (*p* < 0.01). Our study revealed that phi458 promoted biofilm formation by spontaneously inducing and decreasing virulence by repressing virulence genes.

## Introduction

Bacteriophages (phages), naturally occurring viruses that infect bacteria, outnumber bacteria by more than a factor of ten (Brussow and Hendrix, 2002). According to their lifecycles, phages are divided into two groups: lytic phages and lysogenic phages. Lysogenic phages, also called temperate phages, have DNA that is integrated into the bacterial genome or maintained as an episome after infecting bacteria (Feiner et al., 2015). With the advances in whole genome sequencing platforms, it has been proven that most bacterial genomes contain prophage sequences at proportions as high as 20% (Casjens, 2003).

It is worth noting that prophage sequences can also be excised from the bacterial genome when faced with extrinsic factors (e.g., UV radiation, ROS, pH, and heat) or intrinsic factors (stalled replication forks and ROS; Luchnik, 1979). These factors could upregulate the expressions of SOS genes and lead to expression of anti-repressors. The phage repressor protein was cleaved by binding to the antirepressor protein (Crowl et al., 1981). Then, the expressions of lytic phage genes can facilitate prophage excision from the bacterial genome and formation of phage particles (Nanda et al., 2015). Spontaneous prophage induction, an important but uncommon phenomenon, is the spontaneous activation of lytic genes even in the absence of the noninducing factors mentioned above (Santiviago et al., 2010; Nanda et al., 2014).

The influence of spontaneous prophages on bacterial fitness has been studied in a variety of bacterial species. For example, the prophage, Rs551, in *Ralstonia solanacearum*, could reduce the virulence and enhance the competitiveness of its host. Further study found that the presence of the CII phage repressor in Rs551 inhibited the expressions of genes related to twitching motility, extracellular polysaccharide production, and virulence (Ahmad et al., 2017). Similarly, integration of PhiRSM also led to a decline in *R. solanacearum* virulence; the difference was that the CI repressor caused this phenomenon (Addy et al., 2012). In *E. coli* K-12, deletion of all nine prophages resulted in decreased biofilm formation and impaired the ability to adapt to adverse environments. These nine prophages in *E. coli* K-12 were unable to form infectious virus particles, but seven of them were shown to excise spontaneously (Wang et al., 2010). In Shiga toxin-producing *E. coli* (STEC), spontaneous induction of prophages contributed to the entire bacterial population infecting host epithelial cells. Further study found that the type 3 secretion system in STEC, which is required for colonization, is negatively controlled by CII (Robinson et al., 2006; Shimizu et al., 2009).

Avian pathogenic *E. coli* (APEC), a major extraintestinal pathogenic *Escherichia coli* (ExPEC), is the causative agent of avian colibacillosis and causes huge economic loss in the poultry industry worldwide (Kathayat et al., 2021). neonatal meningitis *E. coli* (NMEC) is another important ExPEC which infects the central nervous system of newborns with a high morbidity and mortality (Nielsen et al., 2018). According to comparative genomics and phylogenetic group studies, both APEC and NMEC appeared obvious phylogenetic overlaps and shared some genes related to pathogenicity (for example, salmochelin and type I fimbriae; Ewers et al., 2007; Mitchell et al., 2015). In addition, some highly pathogenic APEC had been proved to be capable of inducing bacteremia or meningitis in mouse models (Tivendale et al., 2010). Therefore, APEC strains are presumed to be an armory of NMEC and be a potential zoonotic bacterium.

Integration of prophages not only brings new genes to the host but also has a stochastic impact on gene expressions (Canchaya et al., 2004). Spontaneous prophage induction may have an impact on the fitness of the bacterial host (Nanda et al., 2015). However, the role of prophages in APEC strains remains largely unknown. Thus, we searched for APEC strains with spontaneous prophage induction *in vitro* and *in vivo*. DE458 was shown to have a high spontaneous rate, and its released phage particles, phi458, were isolated, purified, and sequenced. To investigate the role of this spontaneously inducible prophage phi458 on bacterial fitness, a phi458 deletion mutant was constructed, and the biological properties of the strains were studied.

## Materials and methods

### Bacterial strains and culture conditions

The bacterial strains and prophages used in this work are listed in Table 1. The APEC strain, DE458, was isolated from a duck with clinical neurological symptoms and belongs to the phylogenetic *E. coli* reference (ECOR) Group B2.

**Table 1:** Bacterial strains, phages and plasmids used in this study.

Strain, plasmids or phage	Description	Source
DE458	Wild-type APEC strain isolated, O2 serotype	This study
DE458Δphi458	Phi458 deletion mutant	This study
MC1061	The indicator bacteria	This study
pKD46	Express λ red recombinase, Amp	Datsenko and Wanner (2000)
pKD4	Template plasmid, Kan	Datsenko and Wanner (2000)
pCP20	Yeast Flp recombinase gene, FLP, Cm, Amp	Takara
Phi458	Prophage induced from DE458	Takara

*E. coli* strains were grown overnight at 37°C or 28°C in Luria-Bertani (LB) medium with shaking at 180 rpm. When necessary, the medium was supplemented with appropriate antibiotics [e.g., ampicillin (Amp, 100 μg/ml); kanamycin (Kan, 20 μg/ml); and chloramphenicol (Cm, 30 μg/ml)].

### Screening for spontaneous prophage induction

First, nalidixic acid was used to screen out the strain that could release phage particles under chemical conditions. Chemical phage induction assays were performed as previously described (DeMarini and Lawrence, 1992). Nalidixic acid suspended in sodium bicarbonate solution was sterilized with a 0.22-μm-pore-size membrane filter and added to log-phase growing cultures (OD_600_ = 0.5) at a final concentration of 1 μg/ml. After 3 h of incubation, the cultures were centrifuged at 5000 rpm for 10 min, and the supernatants were collected and passed through a 0.22-μm filter. To confirm the presence of phages in the supernatants, the *E. coli* strain, MC1061, was used as an indicator host for phages, and the double-layer agar method was used.

Second, the strains identified in the previous step were used to screen out the strains that exhibited spontaneous induction phenomena. A single colony of each strain was cultured in LB medium at 37°C for 12 h, and then, the cultured supernatants were collected and filtered. MC1061 was also used as an indicator strain to confirm the presence of phage particles as described previously.

### Phage isolation, purification, and propagation

A single plaque on the double-layer agar plate was picked and incubated in 5 ml of LB medium with the addition of indicator strain MC1061. Phages were purified three times by the double-layer agar method. Finally, SM buffer was added to the double-layer agar plate, which was full of plaques, and incubated 4°C for 12 h. The SM buffer was collected and the bacteria were filtered out with a 0.22-μm filter.

### TEM analysis

Phages were cultured in double-layer plates at 37°C for 10–12 h. A plate with an abundance of plaques was added to 3 ml of doubly deionized H_2_O. Phages were slightly shaken for 10 min to fully wash them off. The suspensions were centrifuged at 5,000 rpm for 10 min, spotted onto a copper grid, and negatively stained with 2% uranyl acetate. The phage morphologies were observed by TEM (H 7650; Hitachi, Japan).

### Induction rates in different environments

To measure the excision rates of phi458 at different temperatures, single DE458 colonies were picked and cultured overnight at 28°C or 42°C for 12 h. To determine the excision rate of phi458 when DE458 interacts with cells, the procedure used was similar to that used for the adhesion assays. DF-1 cells are derived from ELL chicken embryos and are a passable chicken fibroblast cell line which are widely used in bacterial infection model, animal virus research, and many other fields. In summary, chicken embryo fibroblast DF-1 cell monolayers were cultured in sterilized 24-well plates with DMEM (containing 10% fetal bovine serum) at 37°C in a humidified chamber containing 5% CO_2_. Then, the log-phase bacterial cultures were harvested after centrifugation and washing twice with ice-cold DMEM to remove the LB medium and phage particles that had been released. The bacterial suspensions were infected with DF-1 cells (according to an infection multiplicity of 100) for 4 h at 37°C under a 5% CO_2_-humidified atmosphere.

Bacterial DNA under different environmental conditions was extracted and used as a template for qPCR with a one-step qRT-PCR SYBR Green kit (Vazyme). Quantitative real-time PCR was used to determine the prophage excision rates (Lunde et al., 2003). The amounts of bacterial genome that absence of phi458 were measured using the primers flanking phi458 (Table 2); PCR products were generated only if the genome lacked phi458 due to the size of phi458. The relative number of the target gene was normalized to the reference gene, *purA*. Reactions and analyses were performed with an ABI PRISM 7300 Fast Real-time PCR machine.

**Table 2:** Primers used in this study.

Primer	Sequence (5′ to 3′)	Target gene
K1	CAGTCATAGCCGAATAGCCT	pKD4
K2	CGGTGCCCTGAATGAACTGC	pKD4
Kt	CGGCCACAGTCGATGAATCC	pKD4
pKD46-F	GATACCGTCCGTTCTTTCCTT	pKD46-
pKD46-R	TGATGATACCGCCTGCCTTACT	
pCP20-F	ATTGGGTACTGTGGGTTTAGTGGTT	pCP20
pCP20-R	TTGGCTTATCCCAGGAATCTGTC	
Phi458-Mu-F	TGGGACTTGTGAGCGCAGTGTTGATGGGGTAATGCTTTGAATTAGAAGCGGATTCTTATAGTGTAGGCTGGAGCTGCTTC	Phi458
Phi458-Mu-R	TCTGGCACTCTCCGTGCTGGCAGAAGTCGCCTCTGTACGTCTCCATCAGGAGGAGGATTCCATATGAATATCCTCCTTAG	
qPCR-phi458-F	TGGGACTTGTGAGCGCAG	Deletion of phi458
qPCR-phi458-R	TCTGGCACTCTCCGTGCT	
RT-*fimH*-F	CTTATGGCGGCGTGTTATCT	*fimH*
RT-*fimH*-R	CGGCTTATCCGTTCTCGAATTA	
RT-*csgC*-F	CCATTGCTTTGACGAAGTTGAG	*csgC*
RT-*csgC*-R	GCGGCCATTGTTGTGATAAAT	
RT-*eaeH*-F	GTACCCTGAAGGCCACTAAATC	*eaeH*
RT-*eaeH*-R	GACTGCGTTCCGGTAGTAAAG	
RT-*ibeA*-F	ATGACGGTGGGAACAAGAGAA	*ibeA*
RT-*ibeA*-R	ATACCCCTATTGAATCCGCAT	
RT-*aufG*-F	CTGGATCAGCAACCTGGATATT	*aufG*
RT-*aufG*-R	CCCACACATCCGGCATATTA	
RT-*yqiL*-F	AGATCAGACGGTGAACTTTGG	*yqiL*
RT-*yqiL*-R	TCCCGTAATCACATAGCGTAAAT	
RT-*ompA*-F	TGGGTGTTTCCTACCGTTTC	*ompA*
RT-*ompA*-R	GAGTGAAGTGCTTGGTCTGT	
RT-*fepA*-F	CAATGCGCCAGAACATAAAGAG	*fepA*
RT-*fepA*-R	TGTCGAGGTTGCCATACAAG	
RT-*tsh*-F	CACAACCATCCAGGCAGATAA	*tsh*
RT-*tsh*-R	TGTGCCTTCTTCAAGGGTAAA	
RT-*yadC*-F	GGGTCAGGTTCGTTCTTCTC	*yadC*
RT-*yadC*-R	GTGACAGTAGTACCCAGGAATG	

### Isolation of DNA from phage particles for genome sequence analysis

Purified phage particles were collected by PEG-8000 precipitation, and DNA was then isolated from the phage particles by phenol extraction. The extraction of bacterial DNA was performed using a Mega Bacterial DNA Kit (D3350-01) according to the manufacturer’s protocol. The phi458 genomic DNA was sent to Shanghai Weina Biotechnology Co., Ltd. for whole genome sequencing. The next-generation sequencing (NGS) and MiSeq (Illumina) were used for genome sequencing. Raw sequenced reads were filtered for low-quality reads and assembled using Unicycler v 0.43 with a flagged minimum contig length of 1,000 bp. The assembled phi458 genome was annotated using RAST.^1^ VirulenceFinder was used to determine the presence of virulence genes.^2^

### Construction of DE458Δphi458

To investigate the role of phi458 in the DE458 genome, we constructed a DE458 mutant lacking phi458. The phi458 deletion mutant was generated based on the lambda Red-mediated recombination system (Datsenko and Wanner, 2000). First, the flanking regions of phi458 were amplified using plasmid pKD4 as a template with primers. Second, to replace the prophage region, the PCR products were transformed into APEC strain DE458 harboring plasmid pKD46 by electroporation. The mutant was selected on LB plates with kan and confirmed by PCR using primers K1 and K2 combined with primers T-phi458-1 and T-phi458-2. Third, plasmid pCP20 was transferred into the mutant to remove the kan resistance region. Finally, the strain was serially subcultured in LB at 42°C to eliminate plasmid pCP20, which was confirmed by PCR. Thus, we obtained the phi458 deletion mutant and named it DE458Δphi458. The primers used are listed in Table 2.

### Growth curve

Growth was determined by turbidity measurements at 600 nm (OD_600_). Each strain was grown to log phase in LB medium at 37°C. After centrifugation and washing twice with ice-cold PBS, the cells were resuspended in PBS to an OD_600_ of 1.0. Then, 200 μl-suspensions were transferred into Erlenmeyer flasks containing 20 ml of LB. The OD_600_ values were monitored every hour for 16 h. The experiment was performed three times.

### Biofilm formation assays

As previously described (Li et al., 2020), biofilms were determined by crystal violet staining. Briefly, the bacterial suspensions were subjected to an exponential period (OD_600_ = 0.6) and then diluted 1:100 in LB medium. Next, 200 μl aliquots were added to 96-well polystyrene microplates and incubated at 37°C for 36 h. The plates were washed with PBS, and 200 μl of methanol was added to the wells to fix the biofilms for 15 min. After drying at room temperature, the biofilms were stained with 1% (w/v) crystal violet for 15 min. After washing with double-distilled water (ddH_2_O) and drying for 1 h, the stained bacteria were dissolved in 0.2 ml of 95% ethanol for 30 min. The optical densities at a wavelength of 600 nm were determined. Samples were performed in triplicate, and LB medium alone was used as the negative control. The experiments were repeated three times.

### Determination of the median lethal dose (LD_50_)

To assess the virulence levels, 97-day-old chickens were used to measure the 50% median lethal doses (LD_50_) of WT and DE458Δphi458. For each strain, 48 chickens were divided into six groups and challenged intratracheally with 1 × 10^8^ to 1 × 10^3^ CFU of bacteria diluted in 1 ml of PBS. The number of dead chickens was recorded within 7 days, and the LD_50_ values were calculated according to the modified Karber method.

### Adhesion and invasion assays

The adhesion and invasion abilities of the strains were determined as previously described (Fu et al., 2022). For the adherence and invasion experiments, chicken embryo fibroblast DF-1-cell monolayers were cultivated in sterilized 24-well plates with DMEM (containing 10% fetal bovine serum) at 37°C in a humidified chamber containing 5% CO_2_. Then, the log-phase bacterial cultures were harvested, washed twice with ice-cold PBS, and resuspended in DMEM to remove the LB medium. Bacterial suspensions were infected with DF-1 cells (according to an infection multiplicity of 100) for 2 h at 37°C under a 5% CO_2_-humidified atmosphere. For the adhesion assays, after washing three times with ice-cold PBS, the adherent bacteria were lysed with 0.5% Triton X-100 for 10 min and analyzed by the plate counting method. For the invasion assays, the steps were similar to those use for the adhesion assays. After washing out the nonadherent bacteria, the cells were subsequently treated with gentamicin (100 μg/ml) for 1 h to kill the adherent bacteria. The remaining numbers of bacteria that invaded DF-1 cells were determined in the same way as in the adhesion assays. All assays were performed in triplicate and repeated at least three times.

### Analysis of phage cytotoxicity in DF-1 cells

Chicken embryo fibroblast DF-1-cell monolayers were prepared as previously described with some modifications (Henein et al., 2016). DF-1 cells were cultured in 24-well cell culture plates with DMEM (containing 10% fetal bovine serum) at 37°C in a humidified chamber containing 5% CO_2_. When the cells were 80% confluent on the plates, 200 μl of trypsin was added to each well. After 3–5 min of incubation, the cells were suspended in DMEM and centrifuged at 500 × g for 5 min. The supernatants were discarded, and the pellets were resuspended in 2 ml of fresh DMEM.

For trypan blue exclusion assay: DF-1-cell suspensions and 50 μl of purified phage were mixed and incubated in 24-well cell culture plates for 24 h at 37°C under a 5% CO_2_-humidified atmosphere. DMEM and SM were used as the negative controls. After trypsinization, the cell viabilities were measured with Trypan blue assays. In brief, 4 μl of 0.4% trypan blue solution in PBS was added to the cell suspensions, and the cell numbers were immediately counted under an inverted light microscope. Five squares of view were used for each sample. All assays were performed in triplicate and repeated at least three times. For lactate dehydrogenase (LDH) release: LDH values were measured with a colorimetric assay kit (#ab102526, Abcam, United States). These assays were conducted according to the manufacturer’s protocol.

### Bacterial colonization *in vivo*

The strains were cultured to an OD_600_ value of 0.6, washed twice with ice-cold PBS and suspended in an appropriate volume of PBS to ensure that the suspension concentration was 1 × 10^8^ CFU. Two groups of chickens were challenged intratracheally with WT or mutant strain bacterial suspensions (0.1 ml/chicken). At 24 h post-infection, the chickens were euthanized and dissected. The bacterial loads in the lung, heart, and liver were determined by the plate counting method.

### RNA isolation and qRT-PCR

The DE458 and DE458Δphi458 were cultured to an OD_600_ value of 0.6. Then, total RNA was extracted from the bacteria using an E.Z.N.A. bacterial RNA isolation kit (Omega, Beijing, China). HiScript II QRT Supermix (Vazyme Biotech) were used for the cDNA synthesis. The mRNA transcription levels were examined using a One-Step qRT-PCR SYBR Green kit (Vazyme Biotech). The endogenous reference gene *DnaE* was chosen as an internal control, and the 2^−ΔΔCt^ method was used to calculate the mRNA relative expression level. The primers used are listed in Table 2.

### Ethics statement

The chickens used in the study were obtained from a poultry farm in Anhui Province and housed in cages with a 12 h light/dark cycle. The animal study protocol was approved by the Ethical Committee for Animal Experiments of Nanjing Agricultural University (SYXK(SU)2017-0007), Nanjing, China.

### Statistical analyses

The data obtained in this study were analyzed using the GraphPad Prism Software package (GraphPad Software, La Jolla, CA, United States). Mann–Whitney *U*-tests were used to analyze the *in vivo* colonization data. The other data were analyzed by unpaired *t*-tests.

### Accession number

The sequence of phi458 was submitted to GenBank database (accession number OP434518).

## Results

### Screening for APEC strains with spontaneous prophage induction

*Escherichia coli* is divided into four main phylogenetic groups (A, B1, B2, and D), and extraintestinal pathogenic *E. coli* mainly belong to groups B2 and D (Clermont et al., 2000; Zhu Ge et al., 2014; Cordoni et al., 2016). Therefore, 29 strains belonging to B2 or D were selected as screening objects to determine the role of prophages in extraintestinal pathogenic *E. coli*. The results showed that 9 of 29 strains could release phages, and 4 strains exhibited spontaneous induction (Table 3). Among these four strains, DE456 released the largest number of phage particles in LB media (3.5 × 10^5^ PFU/mL), while DE458 was the most after adding Nalidixic acid (4.2 × 10^7^ PFU/mL; Supplementary Table S1).

**Table 3:** APEC strains used in prophage induction experiments.

Strain	ECOR group	Strain	ECOR group	Strain	ECOR group
DE033#	B2	DE139	B2	DE232	D
DE039	D	DE161	D	DE296#*	B2
DE044#	D	DE168	B2	DE297	B2
DE066	D	DE169#*	D	DE298	B2
DE068	B2	DE182#	B2	DE346	B2
DE126	B2	DE183#	B2	DE456#*	D
DE130	D	DE210	D	DE458#*	D
DE142	B2	DE220	D	IMT5155	B2
DE132#	D	DE224	D		
DE134	D	DE231	D		

#Prophage induction of *Escherichia coli* strains after nalidixic acid 1 μg/ml was added. *Phage particles were released by spontaneous induction in LB medium at 37°C.

### The prophage in DE458 had a high spontaneous induction rate *in vivo*

**Figure 1:**
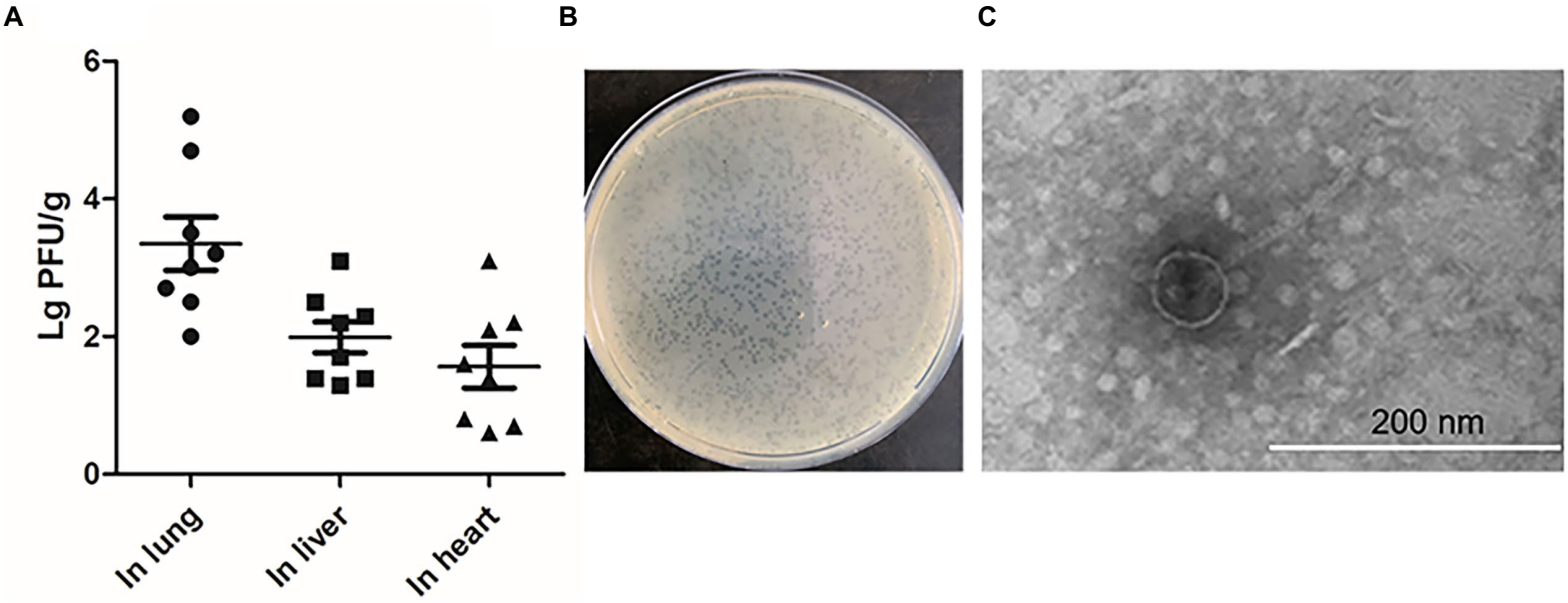
The production of phage during infection *in vivo*. **(A)** The chickens were infected with 5 × 10^6^ CFU of WT DE458 through the air sacs. After 24 h post-infection, phage particles re-isolation from the lung, live and heart were determined by the double-layer agar method. Each data point repressed a sample from an individual chicken. **(B)** Plaque morphology of phi458. **(C)** Transmission electron microscopy (TEM) images of purified phage phi458.

To investigate whether the stains that exhibited spontaneous induction *in vitro* could also release phage particles *in vivo*, systemic infection assays were conducted (Gao et al., 2021). After 24 h post-infection, the numbers of phage particles were measured in the selected organs with the double-layer agar plate method. Among the four strains which exhibited spontaneous induction, no phage particles were detected in liver and heart except for DE458 and the number of phages in lung is shown in Supplementary Figure S1. DE458 had a higher spontaneous induction rate *in vivo* than the other three stains. In particular, maximum colonization was observed in the lung (2.0 × 10^3^ PFU/g), and the lowest colony counts were obtained in the heart (25 PFU/g); the colony numbers in the liver were 100 PFU/g (Figure 1A). The plaques of phi458 showed clear medium-sized plaque spots (0.1 cm, Figure 1B). The transmission electron microscopy results showed that phi458 belongs to the *Siphoviridae* family of long-tailed phages (Figure 1C). Therefore, DE458 was selected as the subsequent research object. To investigate the role of this spontaneously inducible prophage phi458 on bacterial fitness, a phi458 deletion mutant was constructed, and the biological properties of the strains were studied.

### Genomic characterization

**Figure 2:**
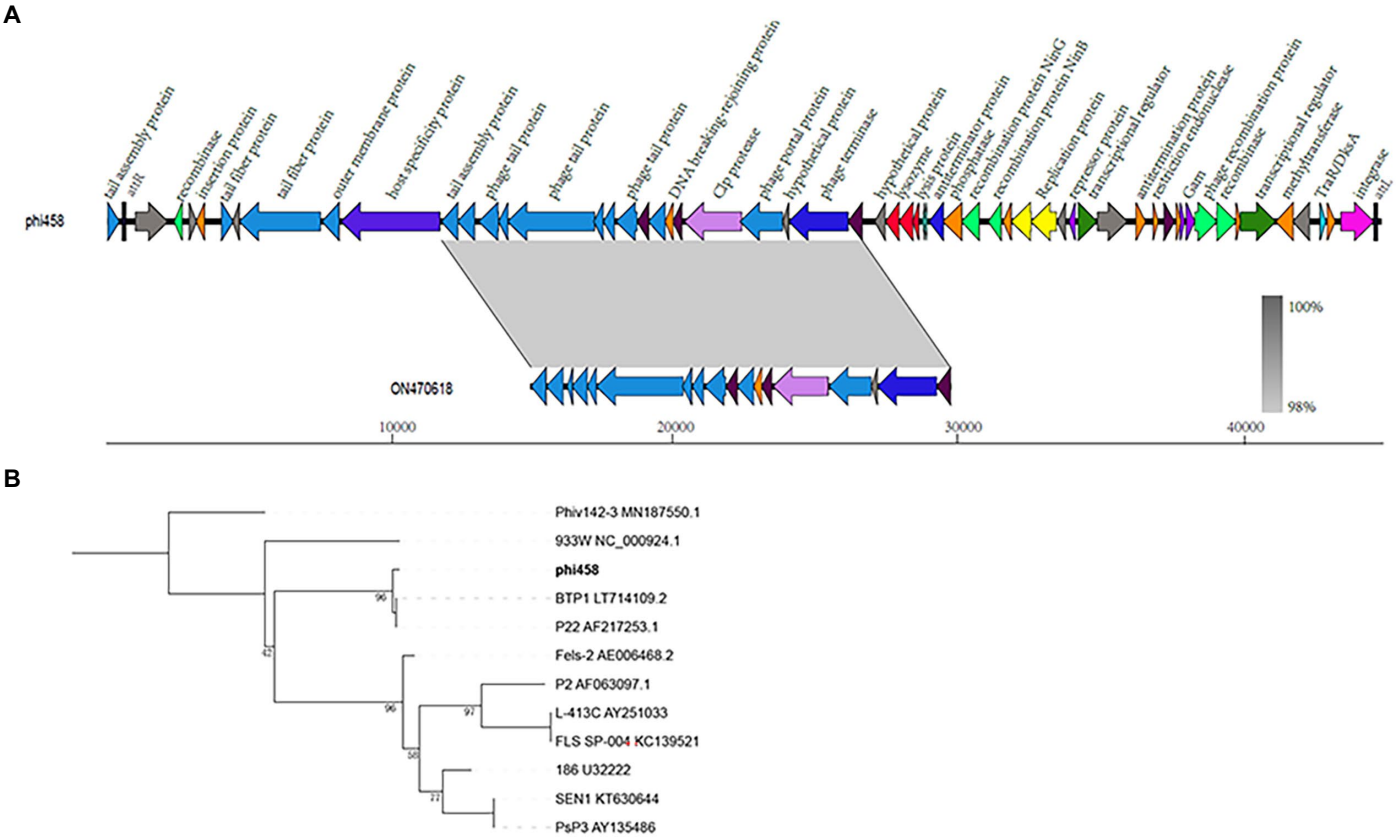
Bioinformation analysis of phi458. **(A)** The structure of prophage phi458. Phi458 is an intact phage and shares a 32% nucleotide sequence identity with *Escherichia* phage vB_EcoS-813R6. The unbroken line represents the length of the sequence. ORFs that are classified in the same functional categories are in the same color. **(B)** Phylogenetic trees of sequence of integrase. Neighbor-joining tree analysis based on the alignment of the amino acid sequence of the integrase. The numbers at the nodes indicate the bootstrap probabilities of that particular branch.

The genome size of prophage phi458 was 44.8 kb, and there were 60 open reading frames, including integrase, lysin, repressor, terminase, portal, protease, tail, transposase, and recombinase. Phi458 shared a 32% nucleotide sequence identity with *Escherichia* phage vB_EcoS-813R6 (Figure 2A). Although in this sequence the similarity was very high, it was regrettable that only have partial genome of *Escherichia* phage vB_EcoS-813R6 (14.9 kb) in NCBI and could not find more information for analysis.

Twenty prophages which could transferred into lytic cycle were selected to compare with phi458 (Supplementary Table S2). As more than half of them shared the integrase gene, the phylogenetic trees obtained using the integrase sequence exhibited similar topologies and supported assignment of phi458 to a sublineage shared by *Salmonella* phage BTP1 and *Salmonella* phage P22 (Figure 2B).

### The spontaneous induction rates of DE458 increased when incubated with DF-1 or grown at 42°C.

**Figure 3:**
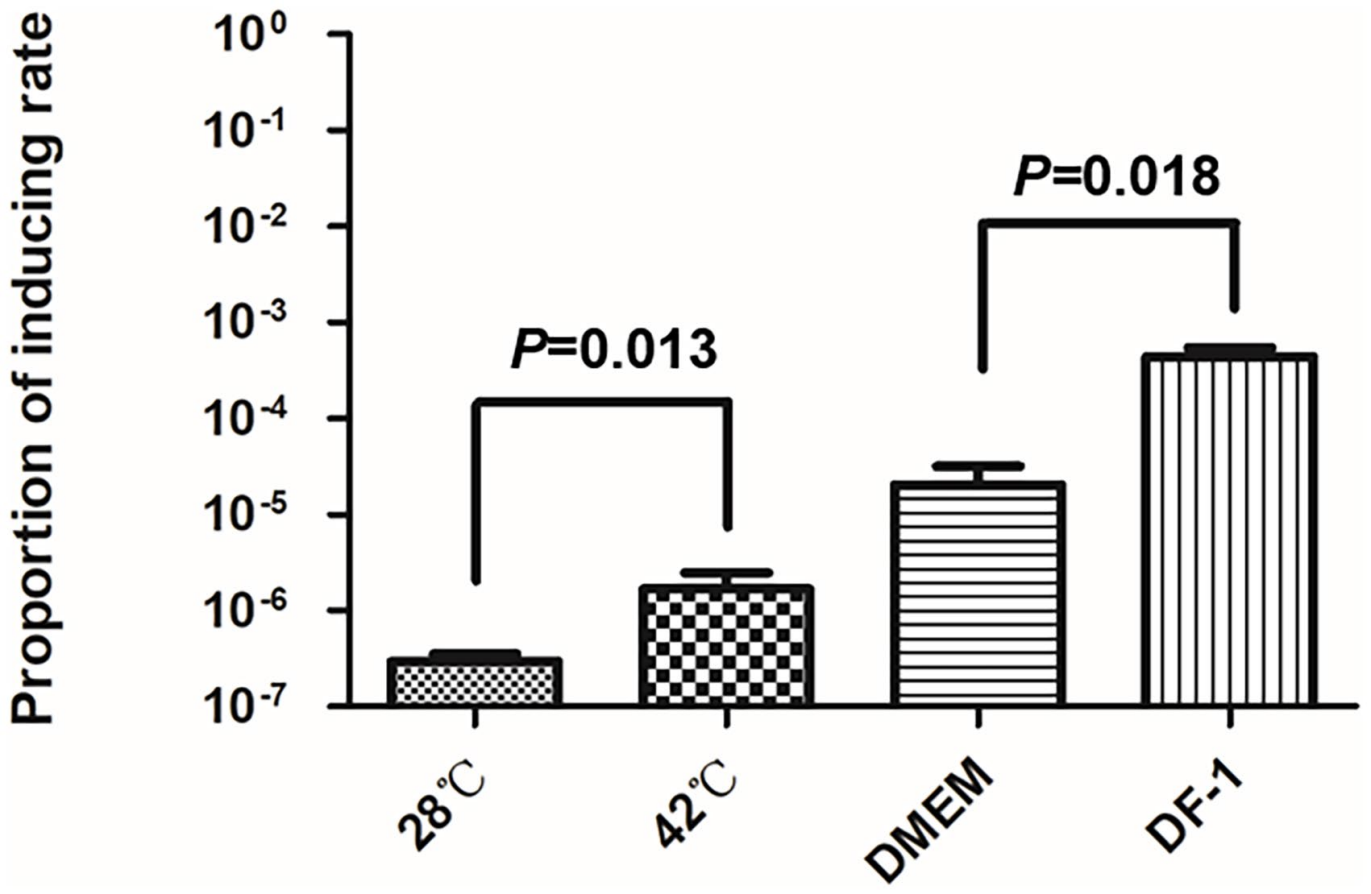
Prophage inducting rate in different environment. Quantitative real-time PCR was used to determine the prophage excision rate. The amount of bacterial genome that was lost of phi458 was measured using primers flanking of phi458, it only gave PCR products if the genome lack of phi458. The relative number of the target gene was normalized to reference gene *purA*. The columns represent the means and standard deviations of three experiments. Unpaired *t*-test were performed for significance.

As the body temperature of chickens is around 42°C, we measured the spontaneous inductions rates at 28°C and 42°C by qPCR. As shown in Figure 3, compared with the induction rate of DE458 at 28°C, the induction proportion was increased by 1.5-fold at 42°C. Furthermore, when DE458 was incubated with DF-1 cells, the induction proportion increased from 2 × 10^−5^ to 4 × 10^−4^ (*p* < 0.01). The results indicated that higher temperatures and interactions with host cells could promote phi458 release.

### Phi458 contributed to biofilm formation of APEC strain DE458

**Figure 4:**
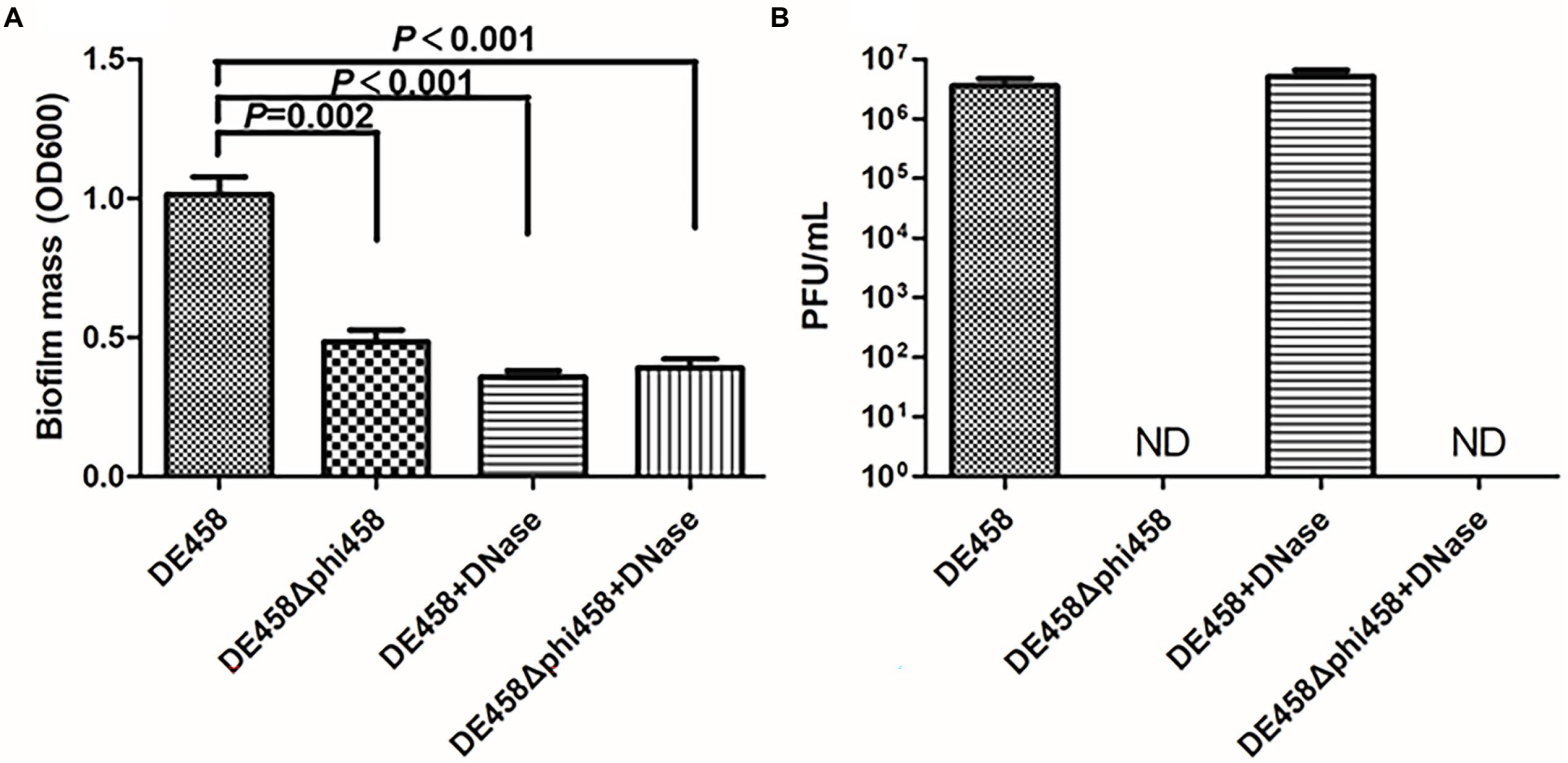
Phi458 increased biofilm formation. **(A)** Biofilm formation abilities of DE458 and its mutant strain DE458Δphi458. The biomass of each strain was measured in the absence or presence of DNase for 36 h. **(B)** The concentration of phage particles phi458 in the supernatant. After 36 h cultivation, the phage in supernatant was filtrated and calculated by the double-layer method. Data are the averages of six replicate wells in 96-well plates from three independent experiments. Error bars indicate standard deviations. Unpaired *t*-test were performed for significance.

Biofilm formation is an important factor in pathogenic bacteria and is crucial for bacteria to resists in nature (Klemm et al., 2010; Gao et al., 2019). Thus, the ability of strains to form biofilms was measured in 96-well microtiter plates. As shown in Figure 4A, biofilm formation of DE458Δphi458 showed a strong decrease compared to that of the WT strain (*p* < 0.01). In addition, while addition of DNase (100 μg/ml) did not affect prophage release but could digest eDNA, it significantly reduced the biofilm production of DE458 biofilm to a level close to that of DE458Δphi458. DE458Δphi458 biofilms were not significantly affected after addition of DNase. Furthermore, phi458 could be induced in mature biofilms at a concentration of 5 × 10^6^ PFU/mL, while no phages could be detected in DE458Δphi458 (Figure 4B). These data suggested that induction of prophage phi458 in DE458 contributes to biofilm formation by releasing eDNA.

### Deletion of phi458 increased adhesion and invasion

**Figure 5:**
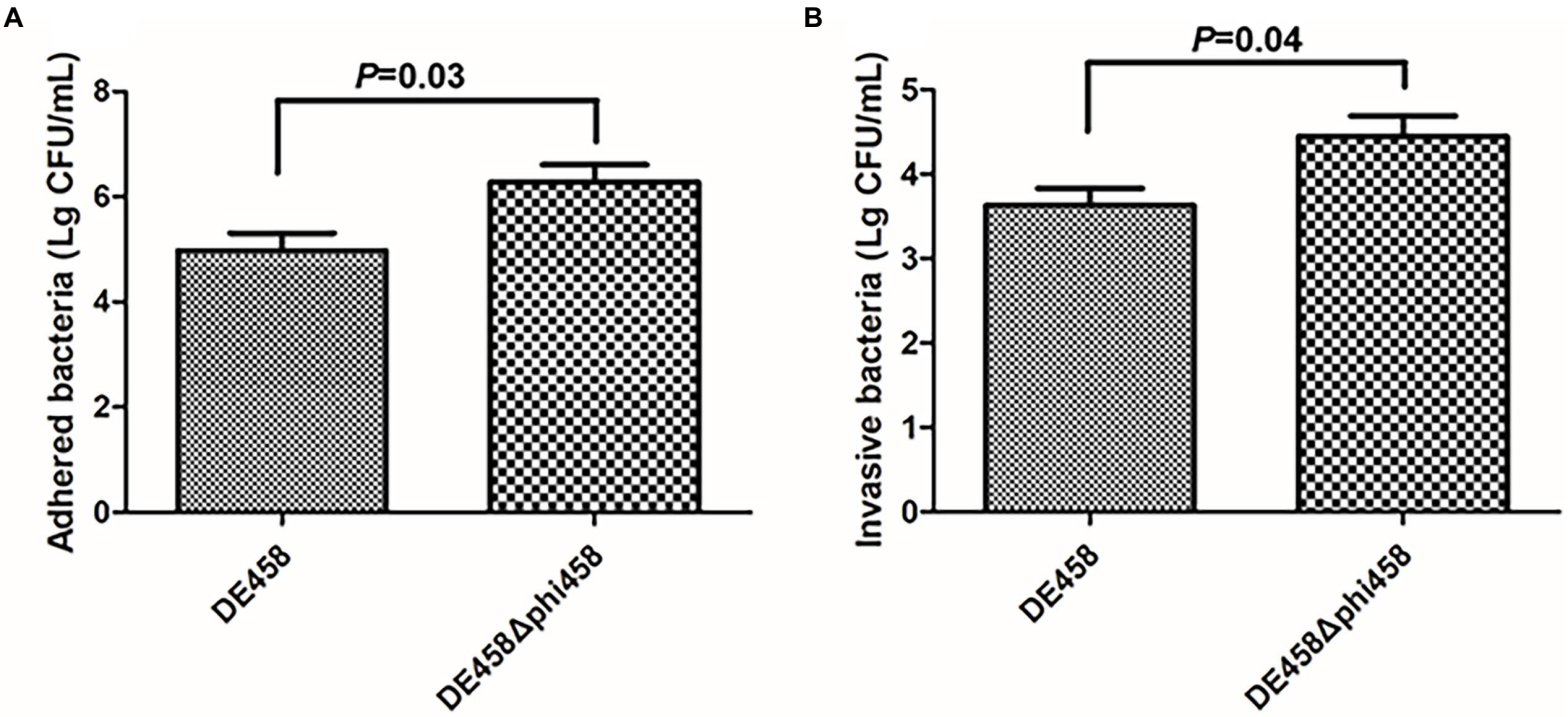
**(A,B)** Adherence and invasion assay. Both adherence and invasion of DF-1 cells by the mutant were significantly increased, as compared to the wild-type strain DE458. Values are the average of three independent experiments. Error bars indicate standard deviations. Unpaired *t*-test were performed for significance.

To assess the effects of phi458 on adhesion and invasion, assays were conducted on the DF-1-cell line. Compared with the WT strain, the numbers of DE458Δphi458 that adhered to DF-1 cells increased from 9.4 × 10^4^ CFU/ml to 1.9 × 10^6^ CFU/ml, while the amounts of invasive bacteria increased from 4.2 × 10^3^ CFU/ml to 2.8 × 10^4^ CFU/ml (Figure 5). In summary, the ability of the mutant strain to adhere to and invade DF-1 cells increased by approximately 6–20 times (*p* < 0.05 by unpaired *t*-tests).

### Deletion of phi458 enhanced the virulence of APEC strain DE458

To investigate the effect of phi458 *in vivo*, chickens were used to evaluate the LD_50_ values of DE458 and its mutant strains. The chickens were challenged intratracheally with the different strains, and the mortalities were recorded daily for 7 days. The LD_50_ values of the phi458 mutant and WT strains were 1.3 × 10^5^ CFU and 1.8 × 10^6^ CFU, respectively (Table 4). The virulence of the wild-type strain was approximately 10-fold weaker than that of DE458Δphi458 (*p* < 0.001). The results indicated that the presence of phi458 led to virulence attenuation in chickens.

**Table 4:** Calculations of LD_50_.

Challenge dose (CFU)	Dead chicks (*n*)/injected chicks (*n*)	
	DE458	DE458Δphi458
1 × 10^8^	8/8	8/8
1 × 10^7^	7/8	8/8
1 × 10^6^	4/8	6/8
1 × 10^5^	2/8	5/8
1 × 10^4^	1/8	2/8
1 × 10^3^	0/8	0/8
LD_50_	1.8 × 10^6^ CFU	1.3 × 10^5^ CFU

**Figure 6:**
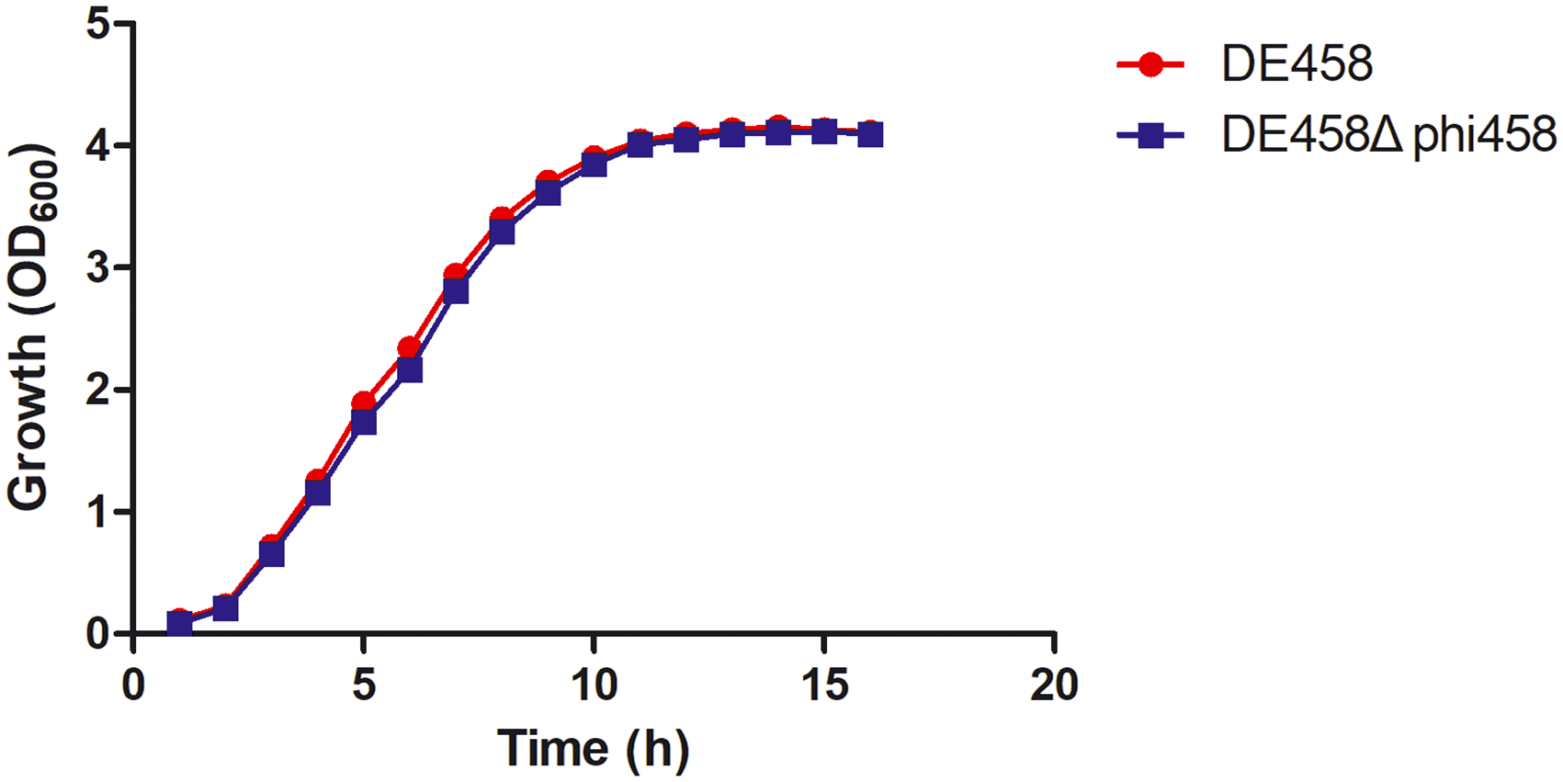
Growth curves of different APEC strains. The wild-type strain DE458 and phi458 mutant strain DE458Δphi458 were grown in LB at 37°C. Growth was determined by measuring the OD_600_. The OD_600_ values were monitored every hour for 16 h. The data represented the average of three independent assays. Unpaired *t*-test were performed for significance.

In order to know if phi458 affects the growth of its carrier strain DE458, the growth curves were determined. The growth curves of the prophage deletion mutant DE458Δphi458 and WT strains did not show significant differences in LB medium (Figure 6), indicating that the increase of virulence in prophage deletion mutant DE458Δphi458 was not due to the change of growth rate.

### Deletion of phi458 increased colonization *in vivo*

**Figure 7:**
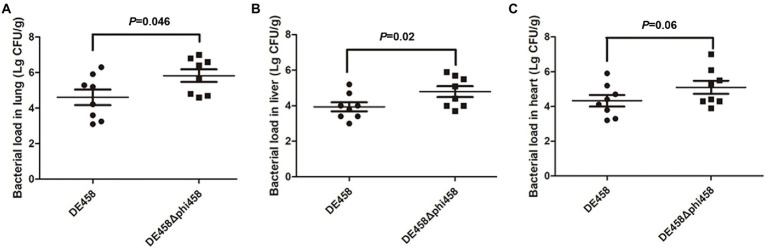
Bacterial colonization during infection *in vivo*. Chickens were changed with strains (5 × 10^6^ CFU for each strain) through the respiratory tract. After 24 h post-infection, the bacterial loads in **(A)** lung, **(B)** liver, and **(C)** hear was counted. Each data point repressed a sample from an individual chicken. The Mann–Whitney *U* test was used to analyze the data.

To assess the effect of phi458 on colonization ability *in vivo*, a systemic infection experiment was performed. At 24 h post-infection, the bacterial loads in selected organs were measured. As shown in Figure 7, the bacterial loads of DE458 in the lung, liver, and heart were 4.0 × 10^4^ CFU/g, 6.3 × 10^3^ CFU/g, and 2.5 × 10^4^ CFU/g, respectively. However, the bacterial loads of DE458Δphi458 were 6.6 × 10^5^ CFU/g (*p* < 0.05), 6.2 × 10^4^ CFU/g (*p* < 0.05), and 1.3 × 10^5^ CFU/g, respectively. The results showed that the colonization ability of DE458Δphi458 increased by approximately 8–40 times compared to that of DE458.

**Figure 8:**
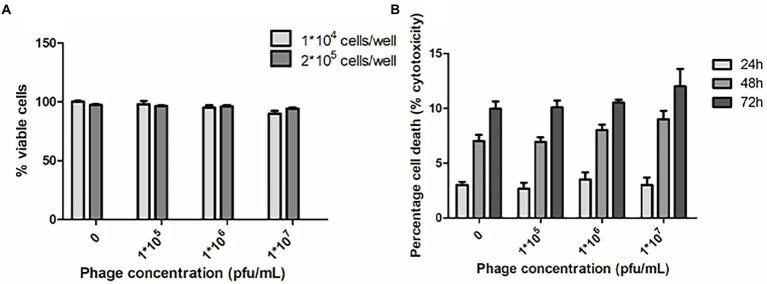
Cytotoxic assays. **(A)** Chicken embryo fibroblast DF-1 cells viability following 24 h exposure to phi458 in DMEM by trypan blue exclusion assay. **(B)** LDH release of chicken embryo fibroblast DF-1 cells following exposure to phi458. Data are the mean of 3 replicates ±SD.

In addition, no genes related to bacterial pathogenicity were found in phi458, as predicted by VirulenceFinder, and phi458 was not cytotoxic to cells (Figure 8), suggesting that phi458 might affect the virulence of DE458 by regulating virulence genes in DE458 rather than itself.

### Deletion of phi458 increased the expression levels of *fimH* and *csgC*

**Figure 9:**
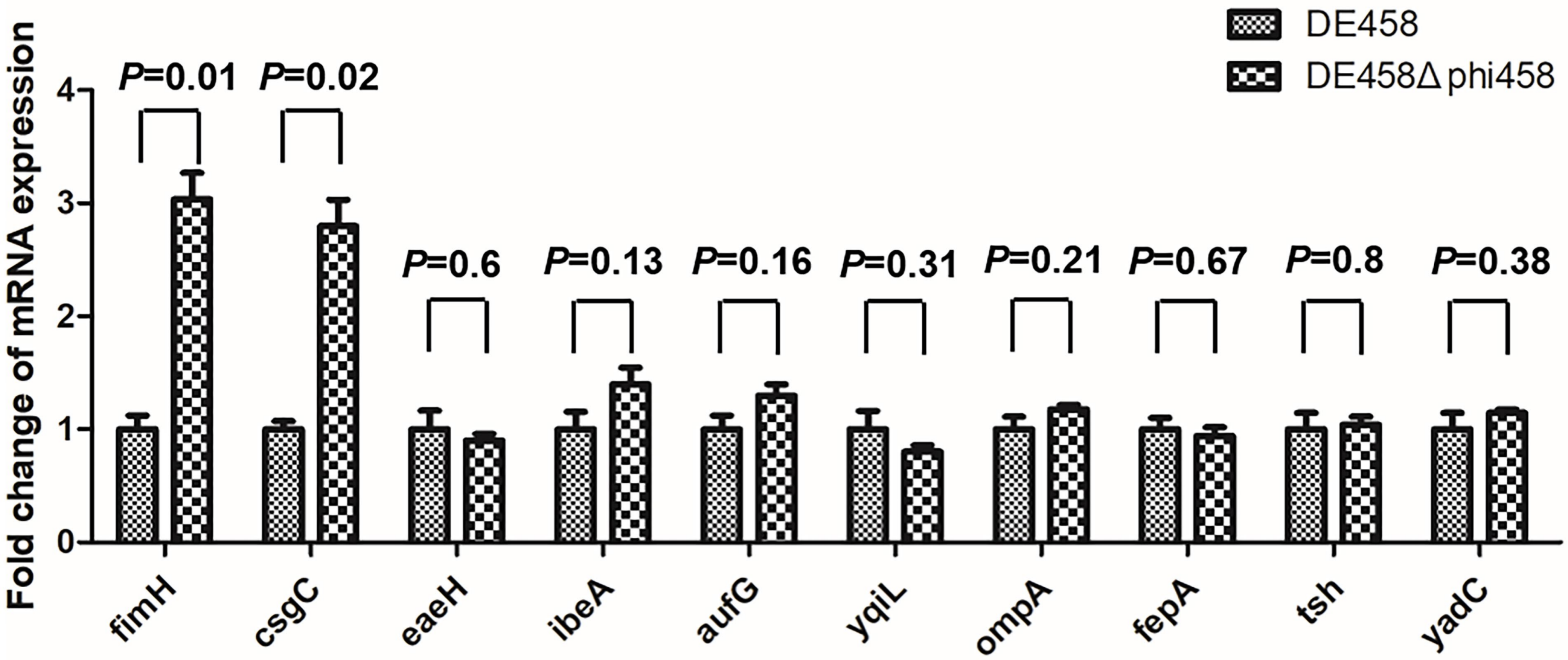
Quantification of virulence genes. The expression level of important virulence genes for APEC infection in strains DE458 and DE458Δphi458 were determined by qRT-PCR. The acquired cycle threshold was normalized to the housekeeping gene *dnaE*. Data were presented as the mean ± SD of three independent experiments. Each experiment is composed of four individual measurements. Unpaired *t*-test were performed for significance.

To investigate the regulatory mechanism of phi458 on the virulence of DE458, the transcription levels of the virulence genes were screened by qRT-PCR. As shown in Figure 9, the transcription levels of *fimH* and *csgC* were upregulated 3-fold and 2.8-fold in DE458Δphi458, respectively. The results suggest that phi458 affected the virulence of DE458 by regulating the expressions of *fimH* and *csgC*.

## Discussion

Currently, the effect of spontaneous prophage induction on the fitness of the bacterial host has been proved in different bacterial strains or species. For example, the phage 933 W could release from STEC, as it carrying exotoxins, the spontaneous induction of phage 933 W facilitates the its bacterial host to infect cells (Livny and Friedman, 2004). The induction of phage DMS3 (in *Pseudomonas aeruginosa* PA14) or Pf4 (in *P. aeruginosa* PAO1) could increase the biofilm formation capacities of the bacterial host (Webb et al., 2003; Zegans et al., 2009). Our study found that APEC strain DE458 had a high spontaneous induction rate *in vitro* and *in vivo*. The released phage particles phi458 were isolated and sequenced. The impact of inducible prophage phi458 on bacterial fitness was investigated in this study.

In nature, bacteria can resist various adverse environments by forming biofilms (Hanlon et al., 2004). As shown in Figure 4A, the biofilms produced by DE458Δphi458 were significantly reduced compared to those produced by the wild-type strain. This result indicated that the presence of phi458 enhanced biofilm formation. Biofilm formation requires various extracellular polymeric substances (Kim et al., 2022), and the spontaneous induction of prophages leads to accumulation of extracellular DNA (eDNA), which contributes to biofilm production (Shen et al., 2018). To test whether a similar mechanism exists in the observed phi458-dependent enhanced biofilm production, DNase was added to the biofilm assay, which significantly decreased the biofilm formation of DE458 to a level close to that of DE458Δphi458. However, the biomass of the DE458Δphi458 biofilm was not significantly changed by the addition of DNase (Figure 4A). Furthermore, phi458 could be induced in mature biofilms at a concentration of 5 × 10^6^ PFU/mL, while no phages could be detected in DE458Δphi458 (Figure 4B). These data strongly indicated that prophage phi458 enhances biofilm production by spontaneous activation. Meanwhile, the phage production provides DE458 with a competitive advantage over a sensitive host (Supplementary Table S1).

To investigate the effect of phi458 *in vivo*, we first examined its induction *in vivo*. Our study showed that phi458 had a high spontaneous induction rate *in vivo* (Figure 1). Extrinsic factors, such as pH, temperature, and organic carbon, can affect the expression level of RecA and induce the SOS response, resulting in the prophage switching to a lytic life cycle (Nanda et al., 2015). In chickens, the body temperature is approximately 42°C, and oxidative stress exists. In order to know which factors may induce prophage phi458 entry into a lytic cycle, we measured the induction rates of DE458 in different environments. The results showed that DE458 had a high induction rate when incubated at 42°C or interacted with DF-1 cells (Figure 3).

An essential step in APEC infection consists of adhesion and invasion of host cells (Klemm et al., 2010). Therefore, we tested the influence of phi458 on the adhesion and invasion ability of DE458. The ability of DE458Δphi458 to adhere to and invade DF-1 cells was increased by approximately 6–20 times (Figure 5). It has been reported that integration of a prophage into a bacterial genome can alter host gene regulation and cause changes in adhesion properties. For example, prophage D3112 in *P. aeruginosa* can inhibit the expression level of the bacterial host gene, *pilB*, which is associated with type IV pilus function and bacterial adhesion ability (Chung et al., 2014). A similar phenomenon was also found in APEC; phiv142-3 regulated the formation of flagella and I fimbriae and contributed accordingly to the adhesion ability (Li et al., 2018).

As previous results suggested that phi458 may affect the colonization ability of DE458 *in vivo*, we determined the colony numbers of mutant and WT strains at 24 h post-infection. As shown in Figure 7, the colonization ability of mutant strain DE458Δphi458 increased compared with that of the WT strain. The results indicate that phi458 affects the colonization ability of DE458. Our main concern is whether phi458 has an effect on bacterial virulence, and we determined the bacterial virulence in chickens. The results showed that the LD_50_ of the mutant strain decreased 10-fold in chickens compared to that of the WT strain, indicating that the integration of phi458 attenuated the virulence of APEC strain DE458.

The prophage affects virulence mainly in two ways: One possibility, prophages can encode some cellular toxins, and release of prophages is a hallmark of several pathogenic bacteria (Goshorn and Schlievert, 1989; Yamaguchi et al., 2000; Sakaguchi et al., 2005). The other possibility, the acquisition and excision of prophages can have an impact on the gene expressions of bacterial hosts, for instance, genes related to motility, EPS production, and virulence (Govind et al., 2009; Rabinovich et al., 2012; Nedialkova et al., 2016). In this study, the cytotoxic effects of phi458 were evaluated in DF-1 cells using two different assays: trypan blue and LDH release. The results showed that phi458 was not cytotoxic to cells (Figure 8). Moreover, no genes related to bacterial pathogenicity were found in phi458. Thus, we hypothesized that phi458 affects the virulence of DE458 by regulating genes related to adhesion and conization.

Virulence alterations by prophages were reported previously. After infection by ϕRSM or Rs551, this could lead to decreased virulence, and the exact mechanism consists mainly of regulating the genes related to virulence or motility by phage suppressor proteins (CI or CII) (Addy et al., 2012; Ahmad et al., 2017). The presence of repressor proteins is necessary to inhibit phage lytic genes to maintain a lysogenic state (Gandon, 2016). Compared with the wild-type strain, qRT-PCR revealed that the transcription levels of *fimH* and *csgC* were upregulated 3-fold and 2.8-fold in DE458Δphi458, respectively (Figure 9). FimH is the adhesin of type I fimbriate (Musa et al., 2009) and csgC is involved in the synthesis of curli (Burall et al., 2004). Both of them contribute to virulence and cell adherence in extraintestinal pathogenic *E. coli* (Antao et al., 2009; Krieger and Thumbikat, 2016). Through bioinformatic analysis (Figure 2), we found that phi458 contains two phage repressor proteins (CI and CII). We speculated that the phage repressor protein in phi458 not only inhibited transcription of the phage lysis module but also repressed the expressions of some virulence genes. Therefore, when DE458 was used to infect animals, the downregulated phage repressor resulted in the prophage switching to the lytic cycle and removed the inhibition of virulence genes. Whether *fimH* and *csgC* are regulated by repressor proteins in phi458 needs further study.

In conclusion, this study reveals that deletion of prophage phi458 leads to decreased biofilm formation *in vitro* and increased colonization ability and virulence *in vivo* by upregulating *fimH* and *csgC* expressions. Our research is first toward a better understanding of relationship between spontaneous induction phage and its carrier APEC by determining the role of prophage phi458 on biofilm formation and virulence of its carrier strain DE458.

## Data availability statement

The datasets presented in this study can be found in online repositories. The names of the repository/repositories and accession number(s) can be found in the article/Supplementary material.

## Author contributions

FT conceived and designed the study. DL was responsible for experimental operation and wrote the manuscript. WL and QH analyzed the data. JR and FX gave experimental help. QL provided valuable suggestions of the manuscript. All authors contributed to the article and approved the submitted version.

## Funding

This work was funded by the National Natural Science Foundation of China (32172858) and China Postdoctoral Science Foundation (grant 2021 M692138).

## Conflict of interest

The authors declare that the research was conducted in the absence of any commercial or financial relationships that could be construed as a potential conflict of interest.

## Publisher’s note

All claims expressed in this article are solely those of the authors and do not necessarily represent those of their affiliated organizations, or those of the publisher, the editors and the reviewers. Any product that may be evaluated in this article, or claim that may be made by its manufacturer, is not guaranteed or endorsed by the publisher.

## Footnotes

^1^ https://rast.nmpdr.org/rast.cgi

^2^ https://cge.cbs.dtu.dk/services/

## References

[ref1] Addy H. S. Askora A. Kawasaki T. Fujie M. Yamada T. (2012). Loss of virulence of the phytopathogen *Ralstonia* solanacearum through infection by phiRSM filamentous phages. Phytopathology 102, 469–477. doi: 10.1094/PHYTO-11-11-0319-R, PMID: 22352303

[ref2] Ahmad A. A. Stulberg M. J. Huang Q. (2017). Prophage Rs551 and its repressor gene orf14 reduce virulence and increase competitive fitness of its *Ralstonia* solanacearum carrier strain UW551. Front. Microbiol. 8:2480. doi: 10.3389/fmicb.2017.02480, PMID: 29312189 PMC5744446

[ref3] Antao E. M. Wieler L. H. Ewers C. (2009). Adhesive threads of extraintestinal pathogenic *Escherichia coli*. Gut Pathog 1:22. doi: 10.1186/1757-4749-1-22, PMID: 20003270 PMC2797515

[ref4] Brussow H. Hendrix R. W. (2002). Phage genomics: small is beautiful. Cells 108, 13–16. doi: 10.1016/S0092-8674(01)00637-7 11792317

[ref5] Burall L. S. Harro J. M. Li X. Lockatell C. V. Himpsl S. D. Hebel J. R.. (2004). Proteus mirabilis genes that contribute to pathogenesis of urinary tract infection: identification of 25 signature-tagged mutants attenuated at least 100-fold. Infect. Immun. 72, 2922–2938. doi: 10.1128/IAI.72.5.2922-2938.2004, PMID: 15102805 PMC387873

[ref6] Canchaya C. Fournous G. Brussow H. (2004). The impact of prophages on bacterial chromosomes. Mol. Microbiol. 53, 9–18. doi: 10.1111/j.1365-2958.2004.04113.x, PMID: 15225299

[ref7] Casjens S. (2003). Prophages and bacterial genomics: what have we learned so far? Mol. Microbiol. 49, 277–300. doi: 10.1046/j.1365-2958.2003.03580.x, PMID: 12886937

[ref8] Chung I. Y. Jang H. J. Bae H. W. Cho Y. H. (2014). A phage protein that inhibits the bacterial ATPase required for type IV pilus assembly. Proc. Natl. Acad. Sci. U. S. A. 111, 11503–11508. doi: 10.1073/pnas.1403537111, PMID: 25049409 PMC4128137

[ref9] Clermont O. Bonacorsi S. Bingen E. (2000). Rapid and simple determination of the *Escherichia coli* phylogenetic group. Appl. Environ. Microbiol. 66, 4555–4558. doi: 10.1128/AEM.66.10.4555-4558.2000, PMID: 11010916 PMC92342

[ref10] Cordoni G. Woodward M. J. Wu H. Alanazi M. Wallis T. La Ragione R. M. (2016). Comparative genomics of European avian pathogenic *E. coli* (APEC). BMC Genomics 17:960. doi: 10.1186/s12864-016-3289-7, PMID: 27875980 PMC5120500

[ref11] Crowl R. M. Boyce R. P. Echols H. (1981). Repressor cleavage as a prophage induction mechanism: hypersensitivity of a mutant lambda cI protein to recA-mediated proteolysis. J. Mol. Biol. 152, 815–819. doi: 10.1016/0022-2836(81)90128-5, PMID: 6460871

[ref12] Datsenko K. A. Wanner B. L. (2000). One-step inactivation of chromosomal genes in *Escherichia coli* K-12 using PCR products. Proc. Natl. Acad. Sci. U. S. A. 97, 6640–6645. doi: 10.1073/pnas.120163297, PMID: 10829079 PMC18686

[ref13] DeMarini D. M. Lawrence B. K. (1992). Prophage induction by DNA topoisomerase II poisons and reactive-oxygen species: role of DNA breaks. Mutat. Res. 267, 1–17. doi: 10.1016/0027-5107(92)90106-c, PMID: 1373845

[ref14] Ewers C. Li G. Wilking H. Kiessling S. Alt K. Antao E. M.. (2007). Avian pathogenic, uropathogenic, and newborn meningitis-causing *Escherichia coli*: how closely related are they? Int. J. Med. Microbiol. 297, 163–176. doi: 10.1016/j.ijmm.2007.01.003, PMID: 17374506

[ref15] Feiner R. Argov T. Rabinovich L. Sigal N. Borovok I. Herskovits A. A. (2015). A new perspective on lysogeny: prophages as active regulatory switches of bacteria. Nat. Rev. Microbiol. 13, 641–650. doi: 10.1038/nrmicro3527, PMID: 26373372

[ref16] Fu D. Shao Y. Li J. Wu J. Wu X. Song X.. (2022). LuxR family transcriptional repressor YjjQ modulates the biofilm formation and motility of avian pathogenic *Escherichia coli*. Res. Vet. Sci. 152, 10–19. doi: 10.1016/j.rvsc.2022.07.011, PMID: 35901637

[ref17] Gandon S. (2016). Why be temperate: lessons from bacteriophage lambda. Trends Microbiol. 24, 356–365. doi: 10.1016/j.tim.2016.02.008, PMID: 26946976

[ref18] Gao Q. Li X. Su S. Yang L. Gao S. (2021). Deletion of the c2515 and c2516 genes affects iron uptake and virulence of APEC O1 strain E516. Front. Vet. Sci. 8:654721. doi: 10.3389/fvets.2021.654721, PMID: 33912608 PMC8075096

[ref19] Gao Q. Xia L. Wang X. Ye Z. Liu J. Gao S. (2019). SodA contributes to the virulence of avian pathogenic *Escherichia coli* O2 strain E058 in experimentally infected chickens. J. Bacteriol. 201:e00625-18. doi: 10.1128/JB.00625-18, PMID: 30602490 PMC6398276

[ref20] Goshorn S. C. Schlievert P. M. (1989). Bacteriophage association of streptococcal pyrogenic exotoxin type C. J. Bacteriol. 171, 3068–3073. doi: 10.1128/jb.171.6.3068-3073.1989, PMID: 2566595 PMC210016

[ref21] Govind R. Vediyappan G. Rolfe R. D. Dupuy B. Fralick J. A. (2009). Bacteriophage-mediated toxin gene regulation in Clostridium difficile. J. Virol. 83, 12037–12045. doi: 10.1128/JVI.01256-09, PMID: 19776116 PMC2786741

[ref22] Hanlon G. W. Denyer S. P. Hodges N. A. Brant J. A. Lansley A. B. Al-Rustamani W. A. (2004). Biofilm formation and changes in bacterial cell surface hydrophobicity during growth in a CAPD model system. J. Pharm. Pharmacol. 56, 847–854. doi: 10.1211/0022357023817 15233862

[ref23] Henein A. E. Hanlon G. W. Cooper C. J. Denyer S. P. Maillard J. Y. (2016). A partially purified *Acinetobacter baumannii* phage preparation exhibits no cytotoxicity in 3T3 mouse fibroblast cells. Front. Microbiol. 7:1198. doi: 10.3389/fmicb.2016.01198, PMID: 27536286 PMC4971803

[ref24] Kathayat D. Lokesh D. Ranjit S. Rajashekara G. (2021). Avian pathogenic *Escherichia coli* (APEC): an overview of virulence and pathogenesis factors, zoonotic potential, and control strategies. Pathogens 10, 467–498. doi: 10.3390/pathogens10040467, PMID: 33921518 PMC8069529

[ref25] Kim U. Kim J. H. Oh S. W. (2022). Review of multi-species biofilm formation from foodborne pathogens: multi-species biofilms and removal methodology. Crit. Rev. Food Sci. Nutr. 62, 5783–5793. doi: 10.1080/10408398.2021.1892585, PMID: 33663287

[ref26] Klemm P. Hancock V. Schembri M. A. (2010). Fimbrial adhesins from extraintestinal *Escherichia coli*. Environ. Microbiol. Rep. 2, 628–640. doi: 10.1111/j.1758-2229.2010.00166.x 23766248

[ref27] Krieger J. N. Thumbikat P. (2016). Bacterial prostatitis: bacterial virulence, clinical outcomes, and new directions. Microbiol Spectr 4:UTI-0004-2012. doi: 10.1128/microbiolspec.UTI-0004-2012, PMID: 26999393

[ref28] Li D. Tang F. Xue F. Ren J. Liu Y. Yang D.. (2018). Prophage phiv142-3 enhances the colonization and resistance to environmental stresses of avian pathogenic *Escherichia coli*. Vet. Microbiol. 218, 70–77. doi: 10.1016/j.vetmic.2018.03.017, PMID: 29685224

[ref29] Li Q. Yin L. Xue M. Wang Z. Song X. Shao Y.. (2020). The transcriptional regulator PhoP mediates the tolC molecular mechanism on APEC biofilm formation and pathogenicity. Avian Pathol. 49, 211–220. doi: 10.1080/03079457.2019.1701182, PMID: 31809574

[ref30] Livny J. Friedman D. I. (2004). Characterizing spontaneous induction of Stx encoding phages using a selectable reporter system. Mol. Microbiol. 51, 1691–1704. doi: 10.1111/j.1365-2958.2003.03934.x, PMID: 15009895

[ref31] Luchnik A. N. (1979). On the mechanism of SOS-repair and prophage induction: relaxation hypothesis. J. Theor. Biol. 77, 229–231. doi: 10.1016/0022-5193(79)90310-2, PMID: 480958

[ref32] Lunde M. Blatny J. M. Lillehaug D. Aastveit A. H. Nes I. F. (2003). Use of real-time quantitative PCR for the analysis of phiLC3 prophage stability in *lactococci*. Appl. Environ. Microbiol. 69, 41–48. doi: 10.1128/AEM.69.1.41-48.2003, PMID: 12513975 PMC152469

[ref33] Mitchell N. M. Johnson J. R. Johnston B. Curtiss R. Mellata M. (2015). Zoonotic potential of *Escherichia coli* isolates from retail chicken meat products and eggs. Appl. Environ. Microbiol. 81, 1177–1187. doi: 10.1128/AEM.03524-14, PMID: 25480753 PMC4292506

[ref34] Musa H. H. He S. F. Wu S. L. Zhu C. H. Liu Z. H. Zhang Z. N.. (2009). Genetic engineering of avian pathogenic *E. coli* to study the functions of FimH adhesin. Indian J. Exp. Biol. 47, 916–920. PMID: 20099466

[ref35] Nanda A. M. Heyer A. Kramer C. Grunberger A. Kohlheyer D. Frunzke J. (2014). Analysis of SOS-induced spontaneous prophage induction in *Corynebacterium glutamicum* at the single-cell level. J. Bacteriol. 196, 180–188. doi: 10.1128/JB.01018-13, PMID: 24163339 PMC3911129

[ref36] Nanda A. M. Thormann K. Frunzke J. (2015). Impact of spontaneous prophage induction on the fitness of bacterial populations and host-microbe interactions. J. Bacteriol. 197, 410–419. doi: 10.1128/JB.02230-14, PMID: 25404701 PMC4285972

[ref37] Nedialkova L. P. Sidstedt M. Koeppel M. B. Spriewald S. Ring D. Gerlach R. G.. (2016). Temperate phages promote colicin-dependent fitness of *salmonella* enterica serovar typhimurium. Environ. Microbiol. 18, 1591–1603. doi: 10.1111/1462-2920.13077, PMID: 26439675

[ref38] Nielsen D. W. Klimavicz J. S. Cavender T. Wannemuehler Y. Barbieri N. L. Nolan L. K.. (2018). The impact of media, phylogenetic classification, and *E. coli* Pathotypes on biofilm formation in Extraintestinal and Commensal *E. coli* from humans and animals. Front. Microbiol. 9:902. doi: 10.3389/fmicb.2018.00902, PMID: 29867813 PMC5951942

[ref39] Rabinovich L. Sigal N. Borovok I. Nir-Paz R. Herskovits A. A. (2012). Prophage excision activates *listeria* competence genes that promote phagosomal escape and virulence. Cells 150, 792–802. doi: 10.1016/j.cell.2012.06.036, PMID: 22901809

[ref40] Robinson C. M. Sinclair J. F. Smith M. J. O'Brien A. D. (2006). Shiga toxin of enterohemorrhagic *Escherichia coli* type O157:H7 promotes intestinal colonization. Proc. Natl. Acad. Sci. U. S. A. 103, 9667–9672. doi: 10.1073/pnas.0602359103, PMID: 16766659 PMC1475797

[ref41] Sakaguchi Y. Hayashi T. Kurokawa K. Nakayama K. Oshima K. Fujinaga Y.. (2005). The genome sequence of clostridium botulinum type C neurotoxin-converting phage and the molecular mechanisms of unstable lysogeny. Proc. Natl. Acad. Sci. U. S. A. 102, 17472–17477. doi: 10.1073/pnas.0505503102, PMID: 16287978 PMC1283531

[ref42] Santiviago C. A. Blondel C. J. Quezada C. P. Silva C. A. Tobar P. M. Porwollik S.. (2010). Spontaneous excision of the *Salmonella* enterica serovar enteritidis-specific defective prophage-like element phiSE14. J. Bacteriol. 192, 2246–2254. doi: 10.1128/JB.00270-09, PMID: 20172996 PMC2849447

[ref43] Shen M. Yang Y. Shen W. Cen L. McLean J. S. Shi W.. (2018). A linear plasmid-like prophage of *Actinomyces* odontolyticus promotes biofilm assembly. Appl. Environ. Microbiol. 84;e01263-18. doi: 10.1128/AEM.01263-18, PMID: 29915115 PMC6102993

[ref44] Shimizu T. Ohta Y. Noda M. (2009). Shiga toxin 2 is specifically released from bacterial cells by two different mechanisms. Infect. Immun. 77, 2813–2823. doi: 10.1128/IAI.00060-09, PMID: 19380474 PMC2708558

[ref45] Tivendale K. A. Logue C. M. Kariyawasam S. Jordan D. Hussein A. Li G.. (2010). Avian-pathogenic *Escherichia coli* strains are similar to neonatal meningitis *E. coli* strains and are able to cause meningitis in the rat model of human disease. Infect. Immun. 78, 3412–3419. doi: 10.1128/IAI.00347-10, PMID: 20515929 PMC2916289

[ref46] Wang X. Kim Y. Ma Q. Hong S. H. Pokusaeva K. Sturino J. M.. (2010). Cryptic prophages help bacteria cope with adverse environments. Nat. Commun. 1:147. doi: 10.1038/ncomms1146, PMID: 21266997 PMC3105296

[ref47] Webb J. S. Thompson L. S. James S. Charlton T. Tolker-Nielsen T. Koch B.. (2003). Cell death in *Pseudomonas aeruginosa* biofilm development. J. Bacteriol. 185, 4585–4592. doi: 10.1128/JB.185.15.4585-4592.2003, PMID: 12867469 PMC165772

[ref48] Yamaguchi T. Hayashi T. Takami H. Nakasone K. Ohnishi M. Nakayama K.. (2000). Phage conversion of exfoliative toxin a production in *Staphylococcus aureus*. Mol. Microbiol. 38, 694–705. doi: 10.1046/j.1365-2958.2000.02169.x, PMID: 11115106

[ref49] Zegans M. E. Wagner J. C. Cady K. C. Murphy D. M. Hammond J. H. O’Toole G. A. (2009). Interaction between bacteriophage DMS3 and host CRISPR region inhibits group behaviors of *Pseudomonas aeruginosa*. J. Bacteriol. 191, 210–219. doi: 10.1128/JB.00797-08, PMID: 18952788 PMC2612449

[ref50] Zhu Ge X. Jiang J. Pan Z. Hu L. Wang S. Wang H.. (2014). Comparative genomic analysis shows that avian pathogenic *Escherichia coli* isolate IMT5155 (O2:K1:H5; ST complex 95, ST140) shares close relationship with ST95 APEC O1:K1 and human ExPEC O18:K1 strains. PLoS One 9:e112048. doi: 10.1371/journal.pone.0112048, PMID: 25397580 PMC4232414

