# Supplementary figures and images for: The effect of a spontaneous induction prophage, phi458, on biofilm formation and virulence in avian pathogenic Escherichia coli
Source: Front Microbiol. 2022 Nov 14;13:1049341. doi: 10.3389/fmicb.2022.1049341 (PMC9701743; doi:10.3389/fmicb.2022.1049341)

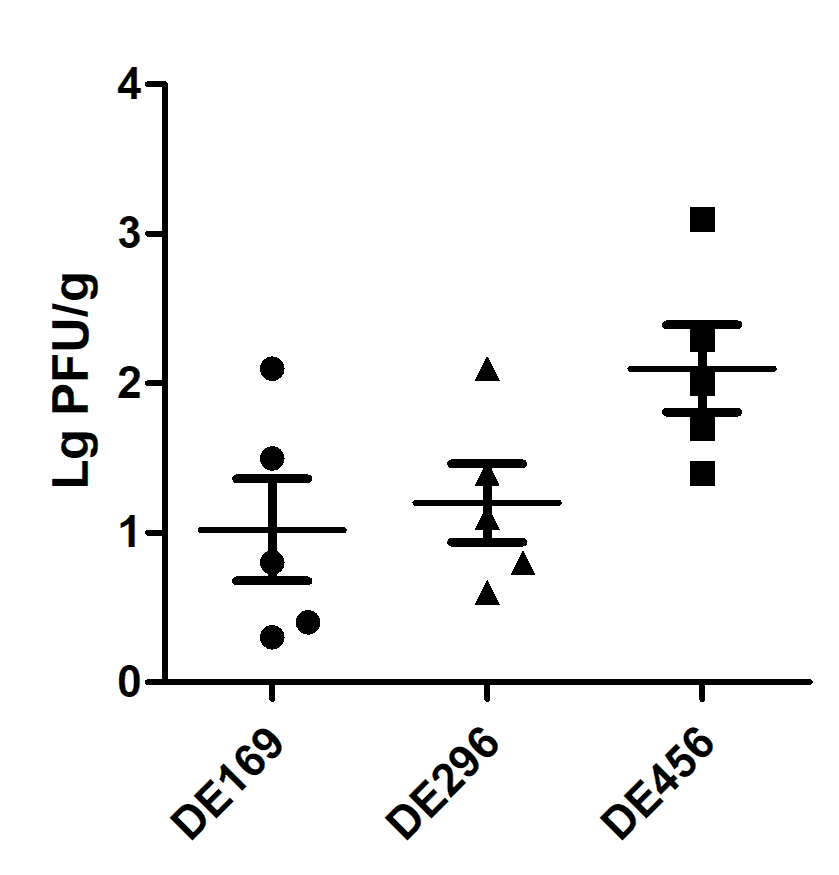

Supplement: Supplementary file 1 [file Image_1.TIF]
