# Supplementary material for: The effect of a spontaneous induction prophage, phi458, on biofilm formation and virulence in avian pathogenic Escherichia coli
Source: Front Microbiol. 2022 Nov 14;13:1049341. doi: 10.3389/fmicb.2022.1049341 (PMC9701743; doi:10.3389/fmicb.2022.1049341)
Supplement: Supplementary file 2 [file Table_1.DOCX]

**Supplementary materials and methods**

**Host range**

A spot assay was used to determine the host range of the harvested phage particle. APEC strains grown to the log phase were spread evenly on an LB plate. A 3 μL liquid containing 10^8^ PFU/mL phi458 was dropped onto the surface of an LB agar plate. Culturing was conducted at 37℃ for 10 h to determine whether there was a lysis zone.
