# Supplementary material for: The effect of a spontaneous induction prophage, phi458, on biofilm formation and virulence in avian pathogenic Escherichia coli
Source: Front Microbiol. 2022 Nov 14;13:1049341. doi: 10.3389/fmicb.2022.1049341 (PMC9701743; doi:10.3389/fmicb.2022.1049341)
Supplement: Supplementary file 3 [file Table_2.DOCX]

**Supplementary Table S1.** The concentration of the released phage in LB media with or without Nalidixic acid

| Strains | Phage released in LB (PFU/mL) | Phage released in LB with Nalidixic acid (PFU/mL) |
| --- | --- | --- |
| DE169 | 1.7×10^3^ | 5.2×10^5^ |
| DE296 | 2.1×10^2^ | 3.6×10^6^ |
| DE456 | 3.5×10^5^ | 1.5×10^7^ |
| DE458 | 1.4×10^4^ | 4.2×10^7^ |
