# Supplementary material for: The effect of a spontaneous induction prophage, phi458, on biofilm formation and virulence in avian pathogenic Escherichia coli
Source: Front Microbiol. 2022 Nov 14;13:1049341. doi: 10.3389/fmicb.2022.1049341 (PMC9701743; doi:10.3389/fmicb.2022.1049341)
Supplement: Supplementary file 4 [file Table_3.DOCX]

**Supplementary Table S2.** Accession numbers and location (for prophage sequence) of phages and prophages used in analysis for Figure 2.

| Phage/prophage | Accession | Host | Location (if prophage) |
| --- | --- | --- | --- |
| SV1 | NC_018848.1 | *Streptomyces* | N/A |
| phiHP33 | NC_016568.1 | *Helicobacter* | N/A |
| LC3 | AF242738.3 | *Lactococcus* | N/A |
| 933W | NC_000924.1 | *Enterobacteria* | N/A |
| DMS3 | NC_008717.1 | *Pseudomonas* | N/A |
| vB_EcoS-813R6 | ON470618.1 | *Escherichia* | N/A |
| P22 | AF217253 | *Salmonella* | N/A |
| BTP1 | PRJEB18919 | *Salmonella* | N/A |
| ϕRs551 | [KX179905](https://www.ncbi.nlm.nih.gov/nuccore/KX179905) | *Ralstonia* | N/A |
| M13 | [NC_003287](https://www.ncbi.nlm.nih.gov/nuccore/NC_003287) | *Enterobacteria* | N/A |
| Phiv142-3 | MN187550.1 | *Escherichia* | N/A |
| Fels-2 | AE006468 | *Salmonella* | 2844421-2879237 |
| P2 | AF063097 | *Enterobacteriaceae* | N/A |
| e14 | CP009273.1 | *Escherichia* | 1195443 - 1210635 |
| CP4-57 | CP009273.1 | *Escherichia* | 2753979 - 2776008 |
| FLS SP-004 | KC139521 | *Salmonella* | N/A |
| SEN1 | KT630644 | *Salmonella* | N/A |
| L-413C | AY251033 | *Enterobacteriaceae* | N/A |
| 186 | U32222 | *Enterobacteriaceae* | N/A |
| PsP3 | AY135486 | *Enterobacteriaceae* | N/A |
